# DSiam-CnK: A CBAM- and KCF-Enabled Deep Siamese Region Proposal Network for Human Tracking in Dynamic and Occluded Scenes

**DOI:** 10.3390/s24248176

**Published:** 2024-12-21

**Authors:** Xiangpeng Liu, Jianjiao Han, Yulin Peng, Qiao Liang, Kang An, Fengqin He, Yuhua Cheng

**Affiliations:** 1 College of Information, Mechanical & Electrical Engineering, Shanghai Normal University, 100 Haisi Road, Shanghai 201418, China; xliu@shnu.edu.cn (X.L.); 1000527096@smail.shnu.edu.cn (J.H.); 1000532497@smail.shnu.edu.cn (Y.P.); liangqiao113@163.com (Q.L.); hfq@shnu.edu.cn (F.H.); 2 Shanghai Research Institute of Microelectronics, Peking University, Shanghai 201203, China

**Keywords:** object tracking, DSiam-CnK, template update, CBAM, KCF, SiamRPN

## Abstract

Despite the accuracy and robustness attained in the field of object tracking, algorithms based on Siamese neural networks often over-rely on information from the initial frame, neglecting necessary updates to the template; furthermore, in prolonged tracking situations, such methodologies encounter challenges in efficiently addressing issues such as complete occlusion or instances where the target exits the frame. To tackle these issues, this study enhances the SiamRPN algorithm by integrating the convolutional block attention module (CBAM), which enhances spatial channel attention. Additionally, it integrates the kernelized correlation filters (KCFs) for enhanced feature template representation. Building on this, we present DSiam-CnK, a Siamese neural network with dynamic template updating capabilities, facilitating adaptive adjustments in tracking strategy. The proposed algorithm is tailored to elevate the Siamese neural network’s accuracy and robustness for prolonged tracking, all the while preserving its tracking velocity. In our research, we assessed the performance on the OTB2015, VOT2018, and LaSOT datasets. Our method, when benchmarked against established trackers, including SiamRPN on OTB2015, achieved a success rate of 92.1% and a precision rate of 90.9%. On the VOT2018 dataset, it excelled, with a VOT-A (accuracy) of 46.7%, a VOT-R (robustness) of 135.3%, and a VOT-EAO (expected average overlap) of 26.4%, leading in all categories. On the LaSOT dataset, it achieved a precision of 35.3%, a normalized precision of 34.4%, and a success rate of 39%. The findings demonstrate enhanced precision in tracking performance and a notable increase in robustness with our method.

## 1. Introduction

In the field of information acquisition and processing, visual cues play a predominant role. It is estimated that about 80% of the information that we encounter in daily life is obtained through visual means. With the rapid advancements in computer technology, together with the proliferation of the internet and artificial intelligence, there has been an increased dependency on visual information. Computer vision technology, an extension of human visual and perceptual skills, is proficient in analyzing complex image data. This capability not only reduces eye fatigue but also enhances the efficiency of data processing. Object tracking stands as a pivotal area of study in computer vision, tasked with predicting the location of a target in a video sequence’s current frame based on preceding frames. Central to this is the development of spatial and temporal linkages across consecutive frames. The field has seen considerable growth, driven by advancements in computer hardware and deep learning technologies, leading to its wide application in video surveillance, autonomous driving, and intelligent robotics.

Despite considerable advancements in object tracking research, the field still encounters numerous challenges in real-world applications. These challenges include, but are not limited to, target deformation, scale variations, occlusions, lighting changes, and background distractions. These factors significantly complicate tracking tasks, posing obstacles to enhancing both accuracy and robustness. In the realm of target occlusion, various innovative methods have been introduced, utilizing Kalman filters, particle filters, mean shift, and optical flow. These approaches have been instrumental in establishing a foundation for the ongoing evolution of tracking algorithms.

Object tracking algorithms are composed of single-object tracking (SOT) [1] and multi-object tracking (MOT) [2], depending on the task specifics. MOT involves the concurrent tracking of multiple targets within a video sequence, addressing both target detection and continuous tracking in each frame. These algorithms are adept at discerning the intricate relationships and motion patterns among multiple targets, ensuring the consistent tracking of their trajectories and identities. Contrastingly, SOT algorithms are more focused on precise target matching, yielding high accuracy in positional data. It is worth noting that the progress in SOT plays a crucial role in augmenting the precision of MOT methodologies.

In the field of single-object tracking, algorithms are essentially grouped into three distinct categories. Primarily, traditional methods are rooted in correlation filtering. Bolme et al. [3] introduced the minimum-output sum of squared error algorithm (MOSSE), a seminal development in correlation filtering for single-object tracking. This algorithm, designed to minimize the output sum of squared errors, leverages online learning for enhanced performance. It effectively utilizes training samples to craft a correlation filter and is adept at precisely locating a target’s position in image searches. Henriques et al. [4] utilized the unique features of circulant matrices for sample generation. By integrating these elements with the fast Fourier transform, they notably reduced the algorithm’s computational time. Further, their introduction of kernel mapping led to the development of the circulant structure of tracking-by-detection with kernels (CSK) algorithm, which significantly outperformed the MOSSE algorithm in terms of accuracy. In a subsequent development, they advanced the field of single-object tracking with the introduction of the kernel correlation filter (KCF) [5], based on the minimum mean square error principle. This method utilizes a histogram of oriented gradient (HOG) features for detailed target characterization. It adeptly calculates the correlation between the target template and the search image in the frequency domain, enabling precise and efficient target localization. Building upon the KCF algorithm, Danelljan et al. [6] introduced an advanced approach, the discriminative scale space tracker (DSST) algorithm. This method ingeniously utilizes multi-scale feature maps, effectively addressing the challenges posed by varying target scales, thereby enhancing the algorithm’s adaptability.

The second category encompasses methods grounded in deep learning, such as the accurate tracking by overlap maximization (ATOM) algorithm [7]. This algorithm integrates a target-aware occlusion module, which is adept at creating occlusion maps to enhance target robustness. It achieves this by selectively preserving the target area while occluding the non-target portions, thereby enhancing the algorithm’s proficiency in managing target occlusions. Bertinetto et al. introduced a suite of object tracking algorithms centered on the concept of Siamese networks. In their approach, target and search images are processed through distinct branches of the Siamese network. The pivotal step involves assessing the correlation between the feature maps of each branch, a process that is integral to pinpointing the target’s location within the search image. Among the pioneering applications of Siamese networks in object tracking, the Siamese fully convolutional networks (SiamFC) [8] stand out. These algorithms leverage a fully convolutional neural network for extracting target features. The tracking process is then conducted by analyzing the similarity of these extracted features. Expanding established concepts, the Siamese region proposal network (SiamRPN) [9] integrates a region proposal network (RPN). This methodology involves embedding both the target and search images into a shared feature space within the Siamese network. Object tracking is then accomplished by analyzing the correlation between these images. To overcome the limitations of the SiamRPN algorithm in rendering a detailed segmentation of targets during tracking, the Siamese mask network (SiamMask) [10] was conceived. This innovative approach integrates a specialized segmentation network for occlusions, enabling the creation of precise target maps during the tracking process. Thus, SiamMask stands out by delivering precise segmentation details, aiding in advanced tasks such as target recognition and observing target shape changes. To address the challenges faced by the SiamRPN algorithm due to shifts in target motion patterns, the distractor-aware Siamese region proposal network (DaSiamRPN) [11] was proposed. This algorithm introduces a dynamic Siamese network approach, featuring an adaptive adjustment of its parameters during training. This adjustment enhances the network’s capacity to accommodate variations in the target’s motion, thereby endowing DaSiamRPN with increased robustness and a broader adaptability than static models, especially in dynamic scenarios.

In the realm of object tracking, a third approach integrates the robustness of deep learning with the efficiency of correlation filtering. Valmadre et al. [12] conceptualized the correlation filter network (CFNet). This technique harnesses deep learning for extracting abstract target features from extensive datasets while adeptly applying correlation filter properties to achieve precise target localization in the frequency domain. From a deep learning perspective, Song et al. [13] proposed an end-to-end object tracking model named convolutional residual learning for visual tracking (CREST). This model merges the processes of feature extraction and response generation. It utilizes a single convolutional end-to-end architecture to facilitate collaborative filtering, which is grounded in deep feature analysis.

Siamese neural network-based single-object tracking algorithms are renowned for their remarkable precision and success in tracking individual targets. However, a major issue with most of these algorithms is their excessive dependence on initial frames, leading to an ineffective update of the target template. Consequently, this limits their capability to consistently maintain high accuracy and robustness during the tracking process. This becomes notably problematic in long-term tracking scenarios, particularly when faced with complete occlusions or instances where the target completely exits the frame.

Addressing the issues identified earlier, our research introduces the DSiam-CnK, a Siamese neural network tracking algorithm predicated on template updating. This algorithm is an advancement over SiamRPN, enhanced with the CBAM for improved network structure and amalgamated with the feature template of KCF. It features a dynamic template-updating mechanism, enabling the real-time adjustment of the tracking strategy. Our study contributes the following:

To tackle the limited target recognition in complex scenarios, we have refined our algorithm’s structure by integrating an attention mechanism. CBAM allows the network to selectively focus on informative features while suppressing irrelevant ones. By emphasizing critical regions—such as keypoints or areas that are vital for accurate tracking—CBAM improves the model’s ability to represent the target effectively. In scenarios involving cluttered backgrounds or occluded targets, CBAM enables the model to concentrate on the most relevant features, thus significantly enhancing its robustness and performance in dynamic and occluded tracking situations;

In response to the common limitation of Siamese neural network-based target tracking algorithms, which typically fail to update templates and thus struggle with accurately predicting deformed targets, we introduce a new Siamese neural network tracking algorithm featuring dynamic template updating. This algorithm switches to keypoint-based KCF algorithm prediction when it detects target occlusion, using the motion of visible keypoints as a proxy for the entire target’s movement. Upon target re-emergence, the template is updated to reflect the target’s new state, ensuring continued tracking accuracy;

Our algorithm underwent testing on the OTB2015, VOT2018, and LaSOT datasets, where it was benchmarked against various single-object tracking algorithms. This comparison was aimed at evaluating our algorithm’s advancements in robustness, overall performance, and real-time functionality.

## 2. Related Work

Within the sphere of object tracking, significant advancements have been made by scholars, introducing diverse methodologies and strategies to tackle target occlusion issues. These techniques broadly fall into two categories: conventional object tracking methods and those leveraging deep learning. Moreover, these contributions have not only enriched practical applications but also laid a strong foundation for future research and implementation in the field.

Traditional object tracking methods have retained their significance and applicability in recent years. Particularly favored in environments with resource constraints, such as embedded systems and real-time applications, these methods are preferred for their minimal computational requirements and efficient real-time processing. Additionally, in specific application scenarios and with particular datasets, traditional methods have demonstrated validated performance and continue to offer distinct benefits for solving object tracking problems. Traditional object tracking algorithms are broadly categorized into three types. (1) The Kalman filter and its derivatives: Initiated by Kálmán [14], the Kalman filter is a linear dynamic framework for estimating states. It refines the estimation of a target’s state by iteratively minimizing the discrepancy between predicted and measured values, guided by the dynamic model of the system and actual data. This was followed by the development of the extended Kalman filter (EKF). The EKF is designed to process real-time state estimations and manage noise in nonlinear systems through a process of local linearization, which enhances target tracking efficiency in dynamic contexts. Building upon the EKF, Julier et al. introduced the unscented Kalman filter (UKF) [15], a state estimation technique for nonlinear systems. This method employs a unique set of sampling points to effectively approximate the nonlinear transformations of the system. Within the domain of object tracking, the Kalman filter is essential for predicting the future position or state of a target. It utilizes historical observations and motion models to accurately estimate the target’s imminent state. For video tracking applications, the Kalman filter is key in consistently estimating the target’s position and velocity, enabling effective real-time tracking. (2) Feature matching algorithms: Feature matching algorithms identify key features in images and extract descriptors to ensure consistency of the target across different frames. Commonly used algorithms include scale-invariant feature transform (SIFT) [16], sped-up robust features (SURF) [17], oriented fast and rotated brief (ORB) [18], and binary robust invariant scalable keypoints (BRISK) [19]. Within object tracking endeavors, feature matching algorithms are instrumental in extracting and tracking the target’s unique characteristics by aligning key feature points. These algorithms are particularly effective in tracking in static environments or when the target’s appearance remains largely unchanged. (3) Template matching algorithms: Employing a straightforward technique, template matching involves contrasting a pre-established template with specific areas within an image. This method seeks to identify the region that most closely resembles the template, thereby estimating the target’s location. Methods like normalized cross-correlation (NCC) [20] and the sum of squared differences (SSD) [21] are similarly utilized in object tracking. The process entails selecting a template image to contain the target and then sliding this template across the image to calculate NCC or SSD values at each point. The target’s location is ultimately pinpointed by locating the highest NCC value or the lowest SSD value. In summary, traditional object tracking methodologies mainly utilize manually crafted features or established mathematical models to resolve tracking challenges. Although these methods may have some limitations in complex environments compared to the advanced deep learning techniques, they remain capable of providing reliable outcomes in certain specific situations and tasks.

In recent years, deep learning has largely driven a paradigm shift in object tracking. These methods, with their advanced feature learning and representational capabilities, have opened up new avenues in object tracking. Deep learning models, particularly convolutional neural networks (CNNs) and recurrent neural networks (RNNs), have made significant strides in tracking tasks, e.g., a Siamese network splits the input frame into a search and a candidate region. These regions are then transformed into a feature space by the network. The network calculates the similarity between these regions using metric learning techniques like cosine similarity, which aids in pinpointing the target’s location. The RPN [22] is a prominent deep learning architecture in object detection and tracking. It combines CNN with the technique of region proposal extraction to predict the positions and bounding boxes of targets. In the context of object tracking, RPN efficiently generates candidate regions, which are then utilized for accurate target matching and tracking using deep feature analysis. Deep learning methods, with their advanced capabilities in feature learning and representation, are formidable in the field of object tracking. However, their solitary use can be challenging, particularly in unique or complex scenarios. Consequently, these methods are frequently augmented with other related technologies to maximize performance and robustness. Among the techniques often integrated with deep learning for object tracking are attention mechanisms (see Table 1) and correlation filtering algorithms (see Table 2).

Overall, in the field of object tracking, a multitude of innovative methods have been proposed by researchers to effectively address target occlusion. The integration of technologies such as attention mechanisms, correlation filtering algorithms, and deep learning has led to significant progress, thereby enhancing both the performance and robustness of object tracking algorithms in complex scenarios. The ongoing advancement of these methods enriches the toolbox for object tracking tasks and provides substantial support in solving practical tracking challenges. When utilized individually, both attention mechanisms and correlation filtering algorithms offer distinct benefits. Attention mechanisms focus the model on the target area, enhancing its recognition and tracking, particularly by reducing background noise in complex settings. On the other hand, correlation filtering algorithms, utilizing template matching principles, enable precise target localization, providing both stability and timely performance. However, both attention mechanisms and correlation filtering algorithms come with inherent limitations. The use of attention mechanisms alone may not be effective in situations where the target undergoes appearance changes or is occluded. Similarly, exclusive dependence on correlation filtering algorithms can result in inadequate performance in scenarios involving scale variations or target occlusion.

In an effort to harness the strengths and mitigate the weaknesses of both attention mechanisms and correlation filtering algorithms, this study introduces the DSiam-CnK method, a synergy of channel space attention and correlation filtering. This integration heightens the focus on the target area through attention mechanisms while accurately pinpointing the target’s location using correlation filters, thereby enhancing the algorithm’s efficacy in complex settings. Experimental evidence indicates that this combination substantially enhances performance in a variety of challenging scenarios, highlighting its extensive applicability in object tracking endeavors.

## 3. Methodology

### 3.1. Design of Siamese Network Structure Combined with CBAM

**Figure 1:**
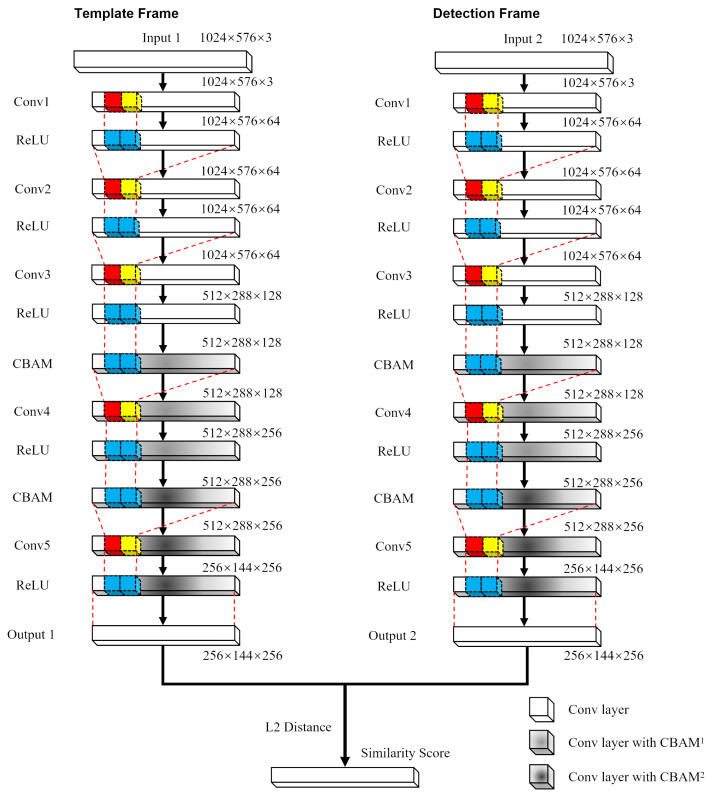
SiamRPN integrated with CBAM.

This section introduces an enhanced version of the SiamRPN algorithm, incorporating spatial and channel attention mechanisms to augment the Siamese network’s efficacy in target representation and tracking precision. The adoption of the CBAM [33] is meticulously calibrated to prevent significant increases in parameter numbers while maintaining the algorithm’s time efficiency. The network architecture of SiamRPN, augmented with the CBAM, is depicted in Figure 1.

To enhance the model’s efficacy, the CBAM is concurrently integrated into both the template and detection branches of the Siamese network. We began by strategically positioning CBAM subsequent to the conv3 and conv4 layers, enhancing the network’s ability to finely tune both channel and spatial attentions. To further boost the capacity for feature representation, an additional CBAM was embedded between the conv4 and conv5 layers, thereby sharpening the model’s acuity in focusing on and learning key features. The implemented design strategy is tailored to substantially enhance the SiamRPN’s perceptual abilities and its efficiency in target detection, ensuring robust support for precise tracking and detection in complex environments. The final stage involves computing the similarity scores derived from the outputs of the two network branches.

**Figure 2:**
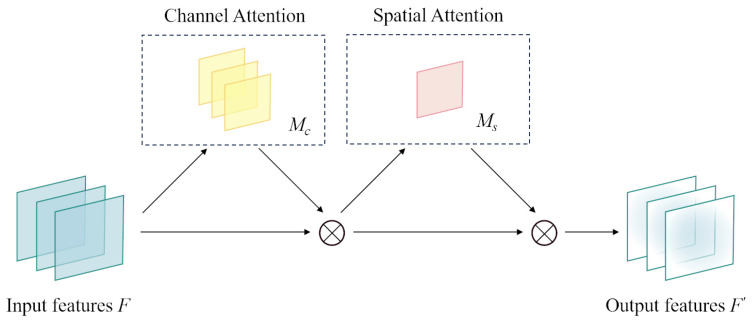
Convolutional block attention module.

As illustrated in Figure 2, the CBAM seamlessly merges both channel and spatial attention modules. Through the dynamic modulation of channel weights and spatial positions within the feature maps, it enables the model to more proficiently discern and exploit crucial features.

Given that the backbone network outputs a feature map *F*, with dimensions H×W and consisting of *c* channels, the CBAM then creates a one-dimensional channel attention feature map $M_{c}\in 1\times 1\times C$ and a two-dimensional spatial attention feature map $M_{s}\in 1\times H\times W$. The procedure for this generation is detailed in the subsequent equations:

(1) $$F^{\prime }=M_{c}\left(F\right)⊗F$$

(2) $$F^{\prime \prime }=M_{s}\left(F^{\prime }\right)⊗F^{\prime }$$

**Figure 3:**
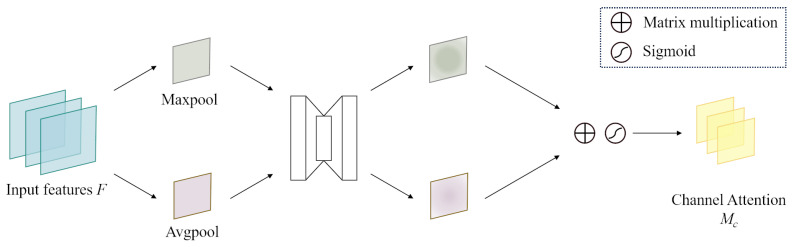
Channel attention modules.

Illustrated in Figure 3, the channel attention module is tailored to integrate attention mechanisms specifically within the feature channel dimension. This module adeptly adjusts the prominence of individual channels within the feature map by applying learned weights. To avoid computational strain from an overabundance of parameters, it efficiently simplifies channel processing using a combination of max pooling and average pooling techniques.

The computational process is outlined as follows:

(3) $$\begin{array}{cc} M_{c}\left(F\right) & =\sigma \{MLP[\mathrm{AvgPool}(F)]+\mathrm{MLP}[\mathrm{MaxPool}(F)]\}\\ & =\sigma \left\{W_{1}\left[W_{0}\left(F_{\mathrm{avg}}^{c}\right)\right]+W_{1}\left[W_{0}\left(F_{\max}^{c}\right)\right\}\right. \end{array}$$

where $F_{\mathrm{avg}\;}^{c}$ and $F_{\max}^{c}$ are representative of the features derived from average pooling and maximum pooling. These descriptors are channeled into a shared network, yielding the channel attention map $M_{c}\left(F\right)$. This network is configured as a multi-layer perceptron with an included hidden layer. After application to each descriptor, an element-wise summation consolidates the output feature vectors. The ensuing step involves element-wise multiplying $M_{c}\left(F\right)$ with F to produce $F^{\prime }$, as delineated in Equation (1).

**Figure 4:**
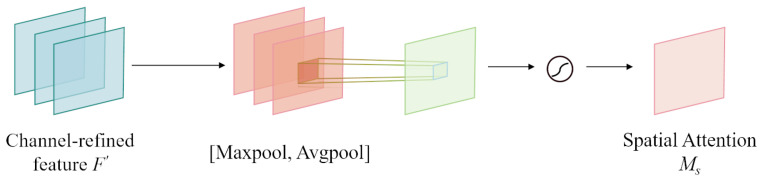
Spatial attention modules.

As illustrated in Figure 4, the spatial attention module is engineered to discern weights across different spatial locations, thereby enhancing the model’s focus on vital regions within the image. To effectively compute channel attention, compressing the spatial dimensions of the input feature maps is essential. For spatial information consolidation, average pooling captures the feature maps’ global context, while max pooling emphasizes the most pronounced features. Consequently, employing both average and max pooling concurrently proves to be instrumental in capturing the spatial details and key features within the feature maps.

In the spatial attention module, channel attention from CBAM is combined with the input feature map *F* to produce an updated feature map $F^{\prime }$ (see Equation (1)). For spatial attention computation, average and global average pooling are applied across the channel dimension, with their outputs concatenated to establish an integrated feature descriptor. Pooling operations with a channel dimension of c are followed by consolidating the channel data of the feature map, and this process results in two distinct 2D feature maps: $F_{\mathrm{avg}\;}^{c}\in \left\{1\times H\times W\right\}$ and $F_{\max}^{c}\in \left\{1\times H\times W\right\}$. These represent the average and max pooled features across channels. These maps are merged and processed through a standard convolution layer, forming the spatial attention map $M_{s}\left(F\right)$. The computational process is as follows:

(4) $$\begin{array}{cc} M_{s}\left(F\right) & =\sigma \left\{f^{7\times 7}\left[\mathrm{AvgPool}\left(F\right);\mathrm{MaxPool}\left(F\right)\right]\right\}\\ & =\sigma \left\{f^{7\times 7}\left[F_{\mathrm{agm}\;}^{s};F_{\max}^{s}\right]\right\} \end{array}$$

Ultimately, an element-wise multiplication of $F^{\prime }$ with the generated $M_{s}\left(F\right)$ is performed, yielding the CBAM’s final output $F^{\prime \prime }$, as shown in Equation (2).

### 3.2. CBAM- and KCF-Enabled Deep Siamese Region Proposal Network

#### 3.2.1. Structure of DSiam-CnK

**Figure 5:**
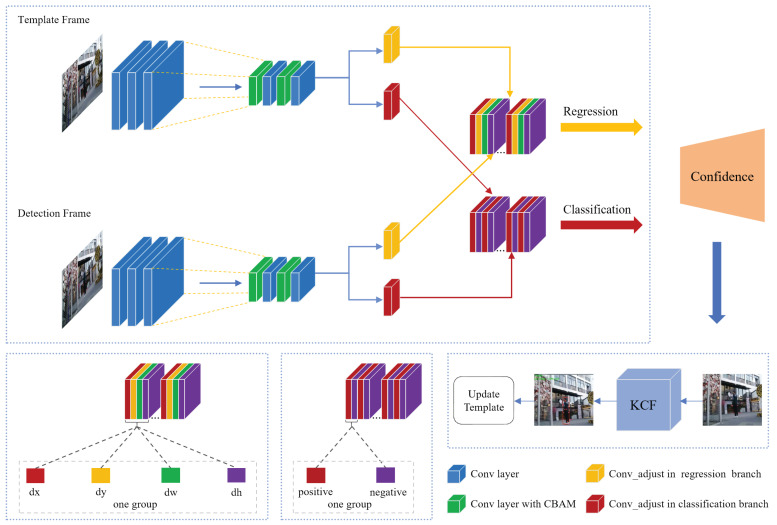
Structure of CBAM- and KCF-enabled deep Siamese region proposal network.

In the prior section, we enhanced the SiamRPN algorithm by integrating it with the CBAM, yielding incremental improvements over its original form, notably under conditions of target occlusion. Motivated by the principles of correlation filtering algorithms, we now propose DSiam-CnK, a Siamese neural network tracking algorithm that melds dynamic template updating with the KCF algorithm (see Figure 5). The essence of this approach lies in leveraging filter templates and feature-point localization to aid the primary tracker, updating the target template within the Siamese network to better accommodate target dynamics, thereby improving both the algorithm’s robustness and its success rate. Following a similar approach, the SiamRPN network was augmented with CBAM subsequent to the conv3 and conv4 layers, thereby enhancing the model’s ability to discern and assimilate essential features. The regression branch processes the candidate target area to output its positional offset (dx, dy, dw, dh), refining the alignment to accurately represent the target’s location. Meanwhile, the classification branch determines whether the candidate area is a target (positive) or background (negative), also generating a confidence score for the target within that area. In instances where the SiamRPN algorithm predicts a low confidence score in the previous frame, the KCF algorithm is applied to forcibly update the target template in the SiamRPN tracker, a crucial step in maintaining tracking accuracy.

The KCF algorithm, introduced by Henriques et al., leverages kernel methods for linear filtering in the Fourier domain, a technique that significantly bolsters the tracker’s precision and resilience. The process begins with the transformation of image patches into feature vectors, followed by the computation of the target template’s circulant matrix within the Fourier domain. The algorithm then proceeds to perform a convolution in this domain, which helps in assessing the similarity distribution between the object under recognition and the target template, thereby enabling precise tracking and localization of the target.

More precisely, the KCF algorithm utilizes ridge regression, aiming to find a function $f\left(z\right)=w^{T}z$ that minimizes the mean squared discrepancy between the sample set $x_{i}$ and the corresponding regression targets $y_{i}$. The formula is as follows:

(5) $$\min_{W}\sum_{i}{\left(f\left(x_{i}\right)-y_{i}\right)}^{2}+\lambda {∥w∥}^{2}$$

where λ serves as the regularization parameter, integral to mitigating overfitting. Consequently, the algorithm’s loss function is formulated as follows:

(6) $$L={\left(f\left(x_{i}\right)-y_{i}\right)}^{2}+\lambda {∥w∥}^{2}$$

When the loss function reaches zero, the corresponding solution is obtained as

(7) $$w={\left(X^{H}X+\lambda I\right)}^{-1}X^{H}y$$

Exploiting the properties of circulant matrix multiplication allows for diagonalizing a circulant matrix with the discrete Fourier transform (DFT) matrix, thereby transforming the matrix inversion problem into an eigenvalue inversion problem. This shift enables the computation for the weight *w* to be conducted in the frequency domain. Enhanced computational speed is achieved by applying DFT, with the solution subsequently being transformed back to the spatial domain to determine the maximal response. Diagonalizing the circulant matrix *X* with the DFT matrix *F* and incorporating it into the preceding formula yields the following result:

(8) $$\begin{array}{cc} w & ={\left(XX+\lambda I\right)}^{-1}X^{H}y\\ & ={\left(F_{\mathrm{diag}\;}\left({\hat{x}}^{*}⊙\hat{x}\right)F^{H}+F_{\mathrm{cliag}\;}\left(\lambda \right)F^{H}\right)}^{-1}X^{H}y\\ & ={\left(F_{\mathrm{diag}\;}\left({\hat{x}}^{*}⊙\hat{x}+\lambda \right)F^{H}\right)}^{-1}X^{H}y\\ & =\left(F_{\mathrm{ciag}\;}\left(\frac{{\hat{x}}^{*}}{{\hat{x}}^{*}⊙\hat{x}+\lambda }\right)F^{H}\right)y \end{array}$$

By leveraging the inverse diagonalization and circulant matrix convolution properties, and expanding the issue into a nonlinear realm through kernel introduction, the regression coefficient *w* is expressed as a linear combination of *x* and the dual space *a*. The regression problem is reformulated as follows:

(9) $$f\left(z\right)=w^{T}z=\sum_{i=1}^{n}a_{i}k\left(z,x_{i}\right)$$

Based on the prior derivation of *w*, when an input image *z* is introduced, regression detection is performed by constructing a kernel correlation matrix between *x* and *z*:

(10) $$f\left(\hat{z}\right)={\hat{k}}^{XZ}⊙\hat{a}$$

A principal innovation of the KCF algorithm lies in its substantial reduction of computational effort in both classifier training and detection phases, a feat achieved through the exploitation of the Fourier diagonalization property of circulant matrices.

#### 3.2.2. Template Update Strategy

The strategy for updating templates in single-object tracking algorithms addresses adapting the target template during tracking to respond to changes in appearance. When the target’s appearance is significantly altered or occluded, most Siamese networks struggle with real-time updates, thereby facing challenges in maintaining target lock. SiamRPN v1 also faces similar challenges: it struggles with substantial appearance changes when the target is observed from different perspectives. Additionally, in scenarios where the target is predominantly occluded, SiamRPN’s ability to track the position is compromised, coupled with a relatively low success rate in re-establishing tracking once it has been lost.

Addressing issues noted in prior research, our approach involves designing an algorithm based on correlation filtering. This algorithm strategically uses visible parts of the human body as tracking points to estimate the target’s position when it is occluded, effectively substituting the movement of these unoccluded features for the entire target frame’s movement. Concurrently, the enhanced SiamRPN algorithm updates a linearly combined template at regular intervals. This template, combining the averaged features of the target from recent frames, adapts to the morphological changes of the target within the video sequence. If the target re-emerges after occlusion, the algorithm compels an update to SiamRPN’s tracking template.

Our experimental investigations of classic correlation filter-based tracking algorithms are directed towards distilling a filtering algorithm that balances high precision, low complexity, and affordability. The findings from these exploratory experiments are summarized in Table 3.

In this study, we integrate the KCF algorithm as a pivotal element for template updating within our algorithm. In integrating the KCF component, we specifically addressed its potential impact on real-time performance while enhancing the model’s adaptability and robustness. KCF utilizes spatial correlations in feature maps, significantly improving the model’s ability to adapt to complex scenarios, such as occlusion and target deformation. This adaptability is crucial for enhancing the stability and robustness of the DSiam-CnK model in dynamic environments. However, as KCF computation relies on frequency domain transformation and feature matching, it may introduce additional latency. To mitigate this, we employed a series of optimizations to control latency while improving robustness, ensuring the model’s real-time capability. Specifically, KCF is triggered for template updates only when occlusion or significant appearance changes are detected, thus avoiding unnecessary computations and reducing latency. Moreover, an adaptive template update mechanism, based on SiamRPN confidence, calls KCF only when confidence falls below a predefined threshold, preventing performance bottlenecks due to frequent updates. Through these optimizations, KCF effectively balances robustness and real-time performance, maintaining latency within reasonable bounds and enabling the model to operate efficiently and stably in dynamic environments.

Initially, a SiamRPN tracker is established for comprehensive target tracking. The methodology then progresses into a repetitive tracking loop, meticulously analyzing each frame of the video. Predominantly, the SiamRPN tracker is employed for global tracking in successive frames. In scenarios where target occlusion occurs, we resort to the KCF tracker for predicting the target’s position, which in turn facilitates the update of the target box within the SiamRPN tracker.

**Figure 6:**
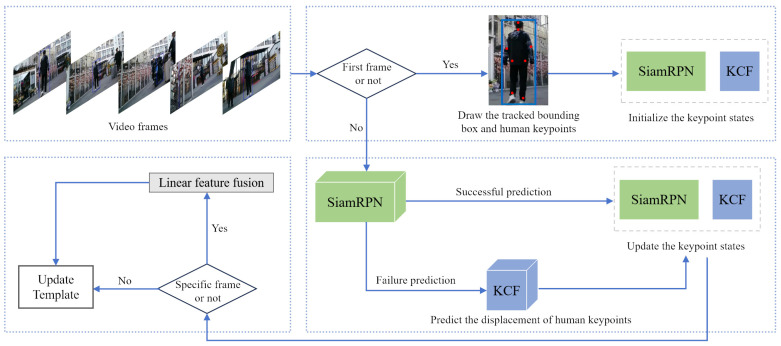
DSiam-CnK algorithm process based on template updating.

In scenarios where the target is not occluded, the SiamRPN tracker is deployed for global tracking. The algorithm enhances accuracy by updating the linear fusion template at fixed frame intervals. If the confidence of the target, initially occluded, reappearing with the current frame’s SiamRPN prediction is low, the program will compulsorily update the SiamRPN tracker’s target template, thereby ensuring precise tracking. For each video frame, the program plots the current tracking box of the target and displays the frame. This sequence is repeated until the video concludes, marking the end of the tracking. The procedure of the Siamese neural network tracking algorithm, predicated on dynamic template updating, is depicted in Figure 6.

The methodology can be explained through the steps in Algorithm 1.
**Algorithm 1** Siamese network tracking with template update steps1:Initialize the SiamRPN tracker.2:Begin the target tracking cycle by utilizing the SiamRPN tracker for comprehensive target tracking.3:**if** In cases of target occlusion, **then**4:Employ the KCF tracker for target position estimation and update the target box in the SiamRPN tracker.5:**end if**6:**if** the target is unoccluded, continue with the SiamRPN tracker. **then**7:Update the template at designated frame intervals (every *N* frames).8:   Update the template if the target, previously occluded, reappears with low prediction confidence in the current frame’s SiamRPN.9:**end if**10:Render and display the tracking box of the current target on each video frame.11:Conclude target tracking.

### 3.3. Selection and Analysis of Datasets

Reliable datasets are essential for advancing algorithms. The research presented in this paper is primarily conducted on three key object tracking datasets: OTB2015, VOT2018, and LaSOT.

**Figure 7:**
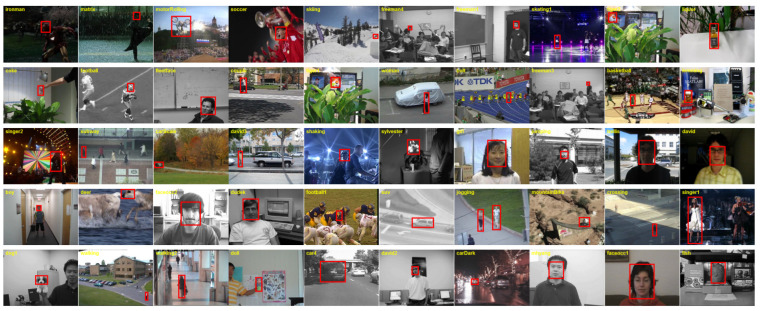
Illustration of the OTB dataset.

The object tracking benchmark (OTB) dataset, a comprehensive collection of video sequences encompassing a range of challenging scenarios, offers a robust tool for evaluating tracking algorithms. It facilitates the uniform comparison and assessment of various tracking methods. OTB2015, with its 50 video sequences, covers diverse scenes, including indoor and outdoor environments and varied lighting conditions, presenting common tracking challenges such as target motion blur, occlusions, and rapid movements. Each frame is precisely annotated with ground truth, clearly marking the target’s position. Evaluations largely depend on the average overlap ratio (AOR), a metric that accurately measures the overlap between the predicted target bounding boxes and their actual positions. Figure 7 illustrates some examples from the OTB dataset.

**Figure 8:**
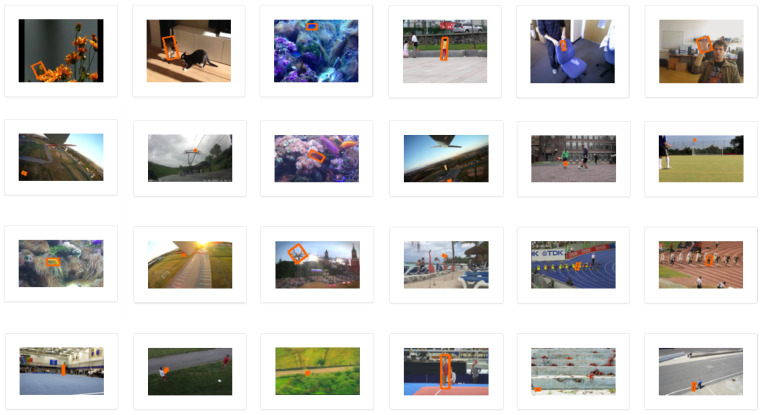
Illustration of the VOT2018 dataset.

The visual object tracking (VOT) dataset, collaboratively established by researchers across various disciplines, stands as a benchmark in the evaluation of visual object tracking algorithms. The VOT dataset has evolved with multiple versions, each offering an extensive collection of varied video sequences. VOT2018, a segment of this series, accentuates real-time and dynamic processing. It encompasses a broad spectrum of scenarios, including rapid movements, scale changes, and target occlusions. Each sequence is meticulously annotated with precise ground truth, highlighting the target’s position within rectangular boxes. For assessment, VOT employs diverse metrics, including tracking accuracy, occlusion duration, and tracking failure count. Figure 8 showcases some instances from the VOT2018 dataset.

**Figure 9:**
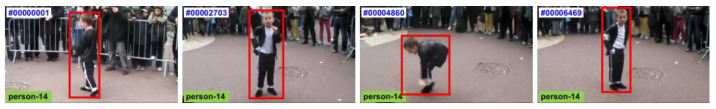
Illustration of the LaSOT dataset.

The large-scale single-object tracking (LaSOT) dataset is a benchmark designed to evaluate the performance of object tracking algorithms, particularly in long-term tracking scenarios. It features 1400 video sequences with over 3.5 million frames, covering a wide range of challenging conditions, including occlusion, background clutter, and scale variations. The dataset is characterized by its significant sequence length, with an average duration of 2500 frames per sequence, making it well-suited for assessing the robustness and accuracy of trackers over extended periods. LaSOT provides detailed annotations, including bounding boxes, target presence indicators, and challenging attributes, enabling comprehensive evaluation and comparison of tracking algorithms. Figure 9 illustrates a subset of the LaSOT dataset.

In essence, OTB2015, VOT2018, and LaSOT are benchmark test sets, specifically crafted to assess the efficacy of object tracking algorithms. They provide an extensive and varied collection of video sequences, facilitating the thorough evaluation of various algorithms across a spectrum of challenging scenarios.

## 4. Experimental Analysis

### 4.1. Comparative Experiments of SiamRPN with CBAM Integration

Initially, the SiamRPN model and its CBAM-enhanced variant, termed SiamRPN-C, are initialized using a pretrained model from the extensive ImageNet dataset. This dataset comprises 1000 categories, with each category containing around 1000 images. Utilizing ImageNet’s pretrained models is beneficial for accelerating the training process, as these models are already trained on a large dataset and possess superior initial weights. Moreover, this approach is efficient in terms of computational resource usage, as the extensive pre-training of these models reduces the need for extensive further training iterations and data.

As for network training, the proposed network undergoes training with a self-annotated dataset, utilizing backpropagation for parameter updates. We apply batch gradient descent for optimization. The initial learning rate is established at 0.005, and the training is conducted over 50 epochs with batches of 8. To prevent overfitting, we set a regularization parameter to 0.0005.

The experimental setup is shown in Table 4.

Renowned in research circles, the OTB dataset serves as a pivotal resource for single-object tracking, featuring a comprehensive collection of video recordings that address a spectrum of tracking challenges. Utilizing this dataset as a foundation, our evaluation of the object tracking algorithm was centered around two principal metrics: the accuracy of target tracking and the precision as quantified by the intersection over union (IoU).

Object tracking accuracy is measured by the Euclidean distance between the tracker’s predicted bounding box and the ground truth box. A smaller distance signifies a higher tracking accuracy, reflecting the tracker’s enhanced precision in localizing the target. For our experimental analysis, the average Euclidean distance is employed as the metric to evaluate tracking precision.

Overlap accuracy quantifies the degree of alignment between the bounding box generated by the tracker and the actual ground-truth box. An elevated overlap rate denotes a closer match of the tracker’s output to the target’s true location, reflecting enhanced tracking accuracy. The IoU metric, representing the ratio of the intersection to the union of the target and ground-truth bounding boxes, is widely adopted for this assessment. Trackers typically yield a series of tracking results over time, necessitating the calculation of tracking precision and overlap accuracy for each frame. The mean of these metrics is then computed to gauge the tracker’s comprehensive performance.

This study was conducted to compare the performances of the SiamRPN and SiamRPN-C models in object tracking tasks and to explore the influence of the CBAM on their effectiveness. The training and testing of both models were conducted on the OTB100 dataset, which consists of 100 video sequences spanning a range of tracking scenarios. Consistent hyperparameters and training approaches were employed for both models. The principal metric for assessment was the AOR, which calculates the mean overlap rate between the target detection box and the actual ground-truth box.

The data presented in Table 5 facilitate a comparative analysis of the SiamRPN and SiamRPN-C models. The integration of the CBAM into the SiamRPN-C model led to enhancements across key metrics: AOR, precision, success, and Euclidean distance. Notably, the AOR metric showed a significant increase, up to 5.5%, indicating that CBAM integration markedly improved the model’s target recognition and tracking effectiveness. CBAM’s adaptive attention mechanism improves the model’s capacity to focus on critical features while suppressing irrelevant background information. This is achieved through the dynamic adjustment of channel and spatial weights, enabling the model to emphasize regions and features that are most relevant to the target, thus enhancing target representation. By embedding CBAM modules in the conv3 and conv4 layers, we aimed to enhance the model’s hierarchical understanding and feature resolution. This integration not only strengthens the model’s focus on the target region but also adapts to variations in target shape and appearance, improving the model’s robustness in handling complex tracking challenges such as occlusion and lighting changes.

These results demonstrate that CBAM contributes significantly to the accuracy and robustness of the SiamRPN-C model, providing a stronger and more precise target localization capability in dynamic scenarios.

### 4.2. Comparative Experiments Among DSiam-CnK and Other Methods

From the analysis in the previous section, it is evident that the SiamRPN-C model exhibits enhancements over the SiamRPN model across a range of performance indicators. Nonetheless, these improvements are somewhat limited, particularly in cases of target occlusion. Motivated by the principles of correlation filtering algorithm-based tracking, this research introduces an innovative tracking algorithm that integrates the KCF algorithm with Siamese neural networks, featuring a dynamic template updating strategy. The essence of this approach is the utilization of filter templates and feature point localization to support the primary tracker, facilitating dynamic template updates within the Siamese network. This method adapts to changes in the target, thereby bolstering its robustness and increasing the success rate.

#### 4.2.1. Data Annotation

**Figure 10:**
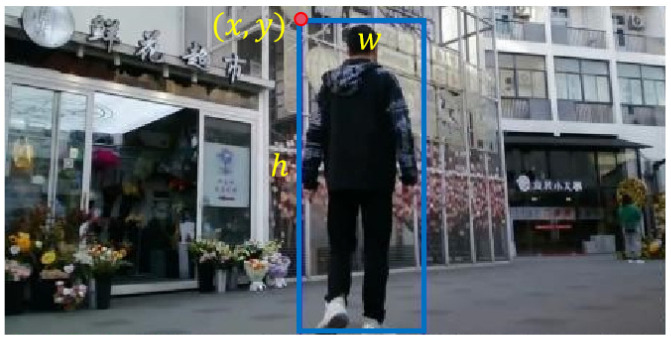
Illustration of template image annotation.

Siamese neural networks operate with dual input sources: the initial frame’s template and search images from the subsequent video sequences. Within the template image *z*, the target’s four parameters (x,y,w,h) are derived, anchored on established actual positional coordinates, as depicted in Figure 10.

For the training process, we utilize template images, labeled as *z*, with dimensions of 127 × 127 pixels, and search images, designated as *x*, of 255 × 255 pixels. Given a bounding box of dimensions (w,h) and background padding of size *p*, the scaling factor *s* is set to ensure that the resultant scaled image retains a consistent size. The formula is as follows:

(11) s(w+sp)×s(h+2p)=A

For the template image *z*, the area is set at $127^{2}$, while, for the search image *x*, it is $255^{2}$. The background padding is determined with a dimension of p=(w+h)/4. To maintain consistency in dimensions, both the template and search images are preprocessed, ensuring no size variations during training.

#### 4.2.2. Loss Function

For the model’s training, the classification loss function, $L_{cls}$, is implemented using cross-entropy, while regression loss, $L_{reg}$, employs the $Smooth_{L1}$ method for coordinate value regression. The dimensions of the actual target bounding box and the corresponding anchor box are represented by $T_{x}$, $T_{y}$, $T_{w}$, $T_{h}$ and $F_{x}$, $F_{y}$, $F_{w}$, $F_{h}$, which denote the center coordinates, as well as the lengths and widths. A subsequent step involves normalizing these parameters, as outlined below:

(12) $${\mathrm{Smooth}}_{L1}\left(x,\sigma \right)=\left\{\begin{array}{cc} \frac{1}{2}\sigma^{2}x^{2} & |x|<\frac{1}{\sigma^{2}}\\ \left|x\right|-\frac{1}{2\sigma^{2}} & \left|x\right|\geq \frac{1}{\sigma^{2}} \end{array}\right.$$

In this context, σ denotes the standard deviation of the input data, allowing the regression loss $L_{reg}$ to be defined as follows:

(13) $$L_{\mathrm{reg}}=\sum_{i=0}^{3}{\mathrm{Smooth}}_{L_{1}}\left(\sigma \left(i\right),\sigma \right)$$

#### 4.2.3. Experimental Evaluation Criteria

The VOT dataset stands as a benchmark for assessing object tracking algorithms’ performance. Beyond traditional metrics like precision, success rate, and robustness, it also incorporates two specialized metrics: expected average overlap (EAO) and expected failure overlap (EFO).

The EAO serves as a comprehensive measure for evaluating a tracker’s overall performance. This metric effectively balances the tracker’s success rate with its precision, providing a more accurate depiction of performance across varied scenarios. The EAO calculation encompasses the tracker’s success rate, precision, and the intricacies of the test sequences, with a higher EAO score indicative of superior tracker performance.

In the context of VOT, the EAO is computed as follows:

(14) $$\hat{\phi }=\frac{1}{N_{h}-N_{1}}\sum_{N_{s}=N_{1}:N_{h}}\hat{\phi }N_{s}$$

where $N_{l}$ and $N_{h}$ denote the lower and upper bounds of a typical interval for video lengths. Moreover, the fraction of videos whose lengths fall within the $N_{l}$ to $N_{h}$ range constitutes 50% of the total video frames.

The EFO serves as a specialized metric, focusing on the tracker’s efficacy during episodes of tracking failure. Essentially, EFO measures the average degree of overlap between the tracker’s bounding box and the true target’s bounding box in instances where tracking is unsuccessful. The EFO value that is lower signifies a tracker’s enhanced performance in scenarios where it fails to track effectively.

#### 4.2.4. Validation and Comparative Experiments

**Figure 11:**
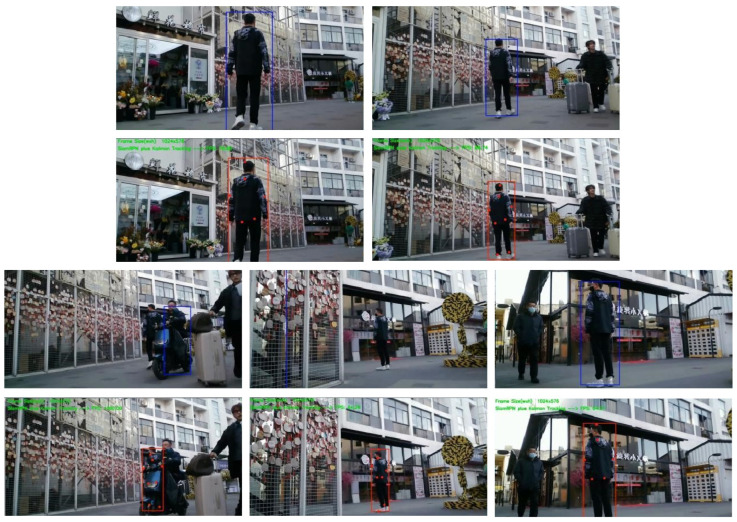
Comparative results: SiamRPN vs. DSiam-CnK.

To assess their performance, the SiamRPN algorithm and its advanced version, DSiam-CnK with dynamic template updating, were compared. The test utilized a self-recorded pedestrian video, which exhibited various tracking challenges, including target scale variation, in-plane rotation, and occlusion. This exercise aimed to preliminarily verify the viability of the proposed algorithm. Figure 11 illustrates the results of this comparison.

Figure 11 showcases frames from five test video sequences, illustrating the outcomes with SiamRPN in blue boxes (above) and DSiam-CnK in red boxes (below).

When occlusion is absent, the first two image sets exhibit comparable results before and after algorithm enhancement. However, as can be clearly observed from the last three sets of comparison images, when an electric vehicle largely occludes the target, and its color and shape are similar to the target, tracking the target becomes challenging. SiamRPN, facing occlusion, often inaccurately shifts its focus to the obstructing object and struggles to reacquire the target in subsequent frames. Conversely, DSiam-CnK effectively copes with extensive occlusion, as clearly demonstrated in the final image set, where it outshines SiamRPN in terms of accuracy.

**Figure 12:**
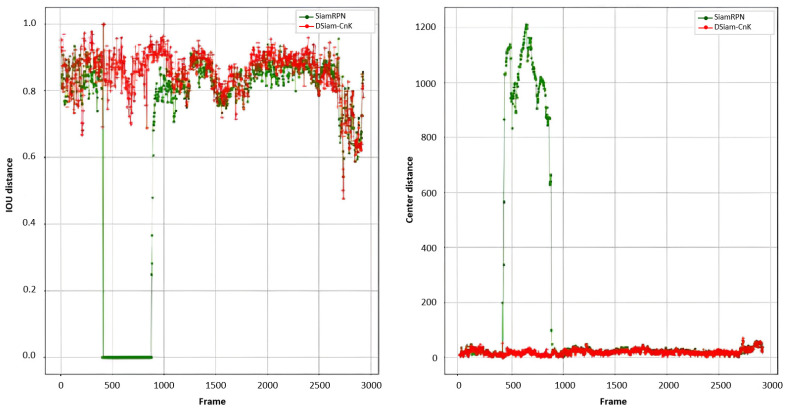
Comparison between SiamRPN and DSiam-CnK: IoU and Euclidean distances.

**Figure 13:**
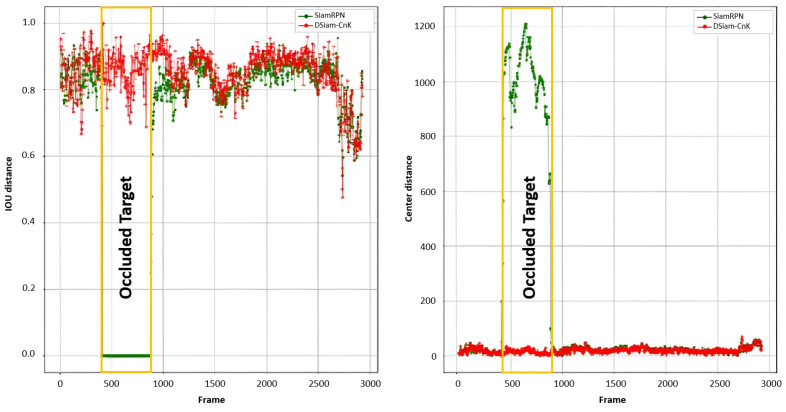
Comparison between SiamRPN and DSiam-CnK: IoU and Euclidean distance variations when the target is occluded (yellow rectangle).

Figure 12 illustrates the comparative outputs of the SiamRPN and DSiam-CnK, across IoU and Euclidean distance metrics. Figure 13 highlights a specific segment of the test video, which spans approximately from the 400th to the 800th frames and is encapsulated in yellow rectangular boxes, depicting scenarios of extensive target occlusion. The comparative analysis reveals that DSiam-CnK markedly excels SiamRPN, as evidenced by its superior overall IoU curve. In instances of target occlusion, SiamRPN’s IoU steeply falls to zero, highlighting its deficiencies in accurately pinpointing the target in such scenarios. In stark contrast, DSiam-CnK demonstrates commendable robustness, maintaining its tracking efficacy amidst occlusions.

With respect to the Euclidean distance metric, the trajectories of DSiam-CnK and SiamRPN align closely when the target experiences minimal occlusion. However, in instances of substantial occlusion, a stark contrast emerges: SiamRPN’s Euclidean distance escalates significantly, while DSiam-CnK maintains a consistent level. This demonstrates DSiam-CnK’s superior capability in tracking targets effectively during occlusion. Such experimental evidence affirms the viability of incorporating correlation filters in Siamese neural networks for template updating, contributing valuable insights for ongoing research in this domain.

### 4.3. Ablation Experiments

To establish the reliability of our DSiam-CnK algorithm, extensive testing was conducted on the OTB2015 and VOT2018 datasets. These two datasets are pivotal in the object tracking domain, with OTB2015 being a standard benchmark and VOT2018 presenting more complex challenges. Their combined use provides a thorough evaluation of the algorithm’s performance under a variety of scenarios.

Experiments conducted on the OTB2015 dataset serve to evaluate the algorithm’s robustness and accuracy across various scenarios. On the other hand, tests on the VOT2018 dataset are geared towards assessing the algorithm’s capabilities in complex and challenging tracking contexts. These comprehensive trials on both datasets are designed to gain a detailed understanding of the proposed DSiam-CnK algorithm’s performance in diverse situations and to confirm its viability and effectiveness in object tracking tasks. In the following sections, we present a detailed overview of the experimental setup, analyze the results, and compare them with those of other algorithms on the OTB2015 and VOT2018 datasets, thereby showcasing the enhanced performance of the DSiam-CnK algorithm.

**Figure 14:**
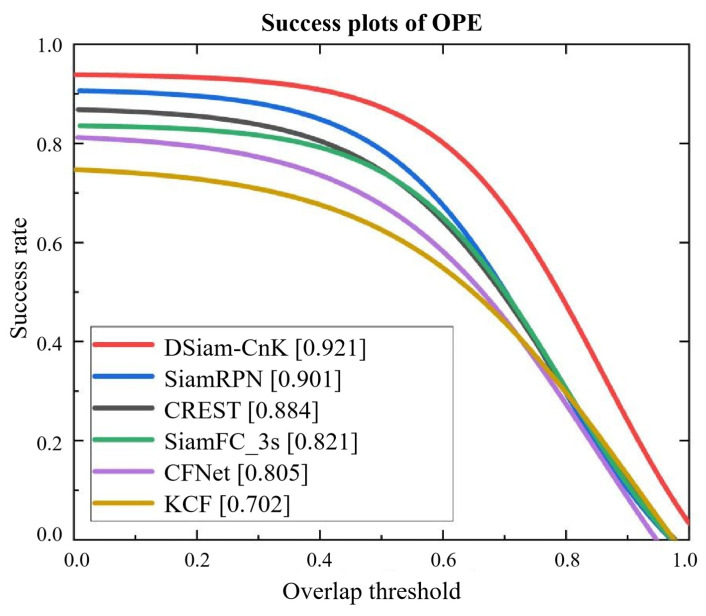
Success plots of OPE.

**Figure 15:**
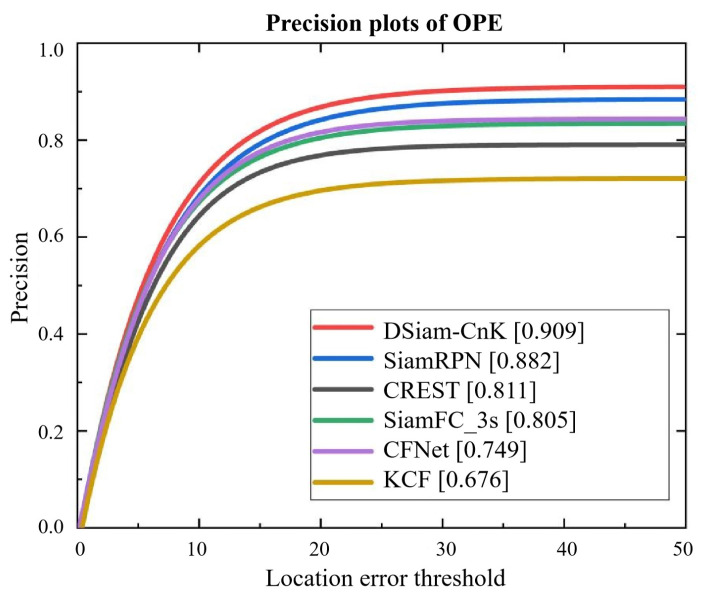
Precision plots of OPE.

To commence our evaluation on the OTB2015 dataset, we compared our DSiam-CnK algorithm, an innovative dynamic template-updating Siamese neural network, with other established algorithms such as SiamFC, SiamRPN, KCF, CFNet, and CREST, which blends correlation filtering with deep learning. The comparative success rates and precision curves of these six algorithms are depicted in Figure 14 and Figure 15, with the OTB2015 dataset as the test set. Detailed data for these comparisons are systematically presented in Table 6. In this analysis, we defined a precision threshold of 45 for final precision metrics and a success rate threshold of 0.1 for final success metrics.

Table 6 lists the performance outcomes of algorithms on the OTB2015 dataset. First, DSiam-CnK, as introduced in this work, surpasses SiamRPN by 3.06% in terms of precision, indicating superior target localization accuracy in tracking tasks. Additionally, DSiam-CnK exhibits a 2.22% increment in success rate, reflecting significant improvements in its tracking stability and reliability. In this comparison, DSiam-CnK distinctly outperforms other trackers like CREST, SiamFC, CFnet, and KCF in both precision and success rate metrics. It surpasses CREST, with an improvement of 12.05% in precision and 4.18% in success rate. When compared with SiamFC, the gains are 12.29% in precision and 11.56% in success rate. Relative to CFnet, DSiam-CnK enhances precision by 21.36% and success rate by 14.41%. Moreover, in comparison to KCF, DSiam-CnK achieves substantial increases of 34.55% in precision and 31.63% in success rate. DSiam-CnK also outperforms SiamR-CNN by 0.22% in precision and 5.29% in success rate, SiamTPR by 0.30% in precision and 5.33% in success rate, and SwinTrack by 0.18% in precision and 4.98% in success rate, demonstrating superior tracking performance across all evaluated metrics.

Presented in Table 7 are the comparative results of algorithms on the VOT2018 dataset. The metrics include precision ‘A’, robustness score ‘R’, and expected average overlap ‘EAO’. DSiam-CnK distinguishes itself in precision, achieving the highest score of 0.467, reflecting its enhanced accuracy in target localization over other methods. In robustness, DSiam-CnK again leads with a score of 1.353, indicating its superior performance. The algorithm also leads in EAO, scoring 0.264, demonstrating its efficacy in complex scenarios, such as handling occlusions and deformations of targets.

Evaluating the results from Table 6 and Table 7, DSiam-CnK demonstrates superior performance over the other five algorithms on the OTB2015 and VOT2018 datasets. Consistently ranking first across various performance metrics, it reveals marked stability and robustness, thus indicating a pronounced edge in target tracking applications.

An analysis of the data in Table 8 validates the effectiveness of the algorithm proposed in this article. When compared with SiamRPN, it demonstrates progress in precision and success rate, with a particularly notable increase in robustness. On the OTB2015 dataset, DSiam-CnK exhibits improvements of 2.22% in success rate and 3.06% in precision over SiamRPN. On the VOT2018 dataset, across the metrics of A, R, and EAO, DSiam-CnK shows enhancements of 2.63%, 33.04%, and 14.29%, respectively. The most pronounced improvement is in the algorithm’s robustness on the VOT dataset, where DSiam-CnK’s robustness score surpasses that of SiamRPN by 33.04%, evidencing its superior performance in handling intricate scenarios like target occlusion and deformation, thereby underscoring its stability and reliability. The results of our experiments demonstrate that the DSiam-CnK tracking approach, developed using a Siamese network with template updates, as proposed in this study, has attained notable enhancements in terms of tracking accuracy and robustness. Especially in the aspect of robustness, the tracking approach introduced in this study outshines other methods, offering greater adaptability and stability.

In Table 9, DSiam-CnK achieves a precision of 0.353, a success rate of 0.390, and a normalized precision of 0.344. It surpasses ECO by 0.052 in precision, 0.066 in success rate, and 0.006 in normalized precision. Compared to DSiam, DSiam-CnK achieves 0.031 higher precision and 0.057 higher success rate, while having a normalized precision 0.061 lower. It also outperforms SiamFC, with a precision increase of 0.014, a success rate increase of 0.054, and a normalized precision decrease of 0.076. When compared to CFnet, DSiam-CnK shows an improvement of 0.094 in precision, 0.115 in success rate, and 0.032 in normalized precision. It also outperforms KCF by 0.187 in precision, 0.212 in success rate, and 0.166 in normalized precision. However, DSiam-CnK is slightly outperformed by MDNet. Overall, DSiam-CnK demonstrates strong and balanced performance on the LaSOT dataset, showcasing its potential to handle challenging tracking scenarios with high precision and robustness.

The performance improvements achieved by DSiam-CnK can be attributed to the integration of a more efficient feature extraction mechanism and enhanced tracking model. The precision boost, particularly in comparison to methods like ECO and KCF, indicates that DSiam-CnK excels at distinguishing targets in challenging scenarios, including occlusions and rapid motion. The relatively slight drop in normalized precision compared to MDNet suggests that while DSiam-CnK performs effectively in real-time tracking scenarios, there may still be room for refinement in handling extreme cases of object deformation and occlusion. The substantial improvements over baseline models like CFnet and SiamFC highlight the effectiveness of the proposed modifications in DSiam-CnK. Furthermore, its robust performance across a variety of tracking tasks underlines its potential as a versatile and reliable solution in real-world applications, such as human tracking, where both accuracy and adaptability are crucial.

## 5. Conclusions and Future Work

Siamese neural networks in target tracking encounter challenges in maintaining accurate tracking when the target is significantly occluded, often resulting in decreased tracking precision or failure. Addressing this issue, this article introduces the DSiam-CnK, which is based on Siamese neural networks and dynamic template updating for target tracking. The principal contributions of this study are outlined as follows:

The paper introduces an attention mechanism into a Siamese neural network for single-object tracking by implementing the CBAM. This enhancement focuses the network on pivotal features, thus substantially boosting the algorithm’s proficiency in discerning targets within complex environments;

Addressing the common shortfall in most Siamese neural network-based tracking algorithms, which typically fail to update templates and, thus, struggle with accurately predicting deformed targets, this study introduces a Siamese neural network tracking algorithm that incorporates dynamic template updating. When encountering target occlusion, it employs a keypoint-focused KCF algorithm for predictions, relying on the motion of visible feature points rather than the entire target frame. Upon the re-emergence of the target, the algorithm adjusts its tracking template to reflect the target’s modifications, substantially enhancing its robustness;

This study involved a comparative analysis of various single-object tracking algorithms on public datasets. The results underscored the enhanced robustness and improved performance of the DSiam-CnK, developed in this work based on dynamic template updating. Notably, the algorithm’s processing velocity substantially exceeds the benchmarks required for real-time applications.

Currently, the method is optimized for SOT. Future research will focus on extending this approach to MOT. The dynamic template-updating mechanism holds significant potential for MOT, as it enables the introduction of a multi-template system, where each object is tracked by an independent dynamic template. Integrating attention mechanisms will aid in distinguishing multiple targets in complex scenarios, enhancing the robustness of the algorithm in the presence of occlusion and target interaction. This extension requires further investigation into template-updating and feature-aggregation techniques to ensure accurate target association in high-density environments.

## Figures and Tables

**Table 1:** Object tracking methods incorporating attention mechanisms.

	Methods	Advantages	Disadvantages	Results
Hua et al. [23]	E-MobileNet extracts features and integrates with a multi-layer fusion network for tracking results.	The method reduces network parameters by nearly a third, significantly enhancing tracking performance.	The algorithm balances speed and accuracy in high-speed motion.	It improves tracking accuracy by 20% to 30% and a real-time tracking speed of 56 FPS on embedded devices.
Chen et al. [24]	The MAM integrates spatial, channel, and temporal attention mechanisms, effectively reducing candidate box numbers and suppressing background noise.	This approach employs LSTM units with an attention mechanism to suppress background noise and highlight target regions.	The method is not real-time; speed enhancements are necessary.	The MAM achieved the highest tracking accuracy at 94.2%, with a nearly 20% increase in speed of processing.
Yang et al. [25]	SiamAtt integrates an attention mechanism into RPN, combining weighted classification and attention branches to pinpoint the final target location.	This framework distinguishes foreground from background in candidate regions using weighted fusion scores.	It is prone to drift during abrupt target changes or occlusions.	SiamAtt achieved an AUC score of 50.3% on the LaSOT dataset, operating at 40 FPS, and attained the highest EAO score of 41.7% on VOT2018.
Wang, C., et al. [26]	The method employs SeNet to enhance extracted features and utilizes LSTM for predicting subsequent positions during target occlusion.	This model enhances feature channel correlation using SeNet, reducing background clutter.	The integration of SeNet and the anti-occlusion module leads to an escalation in both the parameters and the computational demands of the algorithm.	It achieved an accuracy and success rate of 79% on the drone dataset.
Wang, F., et al. [27]	The CSCMOT framework introduced in this method incorporates a non-parametric attention mechanism.	This method enhances real-time performance by focusing on specific target features without increasing computational load.	It employs multi-scale fusion in the baseline model, posing a risk of overfitting.	CSCMOT achieves 32.5 FPS real-time performance with 71.5% accuracy on the MOT17 dataset.

**Table 2:** Object tracking methods incorporating correlation filtering algorithms.

	Methods	Advantages	Disadvantages	Results
Ma et al. [28]	The method learns three correlation filters: translation, scale, and long-term, using their output responses to discern tracking failures.	It employs HOG and HOI features to learn correlational filters, enhancing localization precision.	The approach’s separate use of translation and scale filters may result in information loss.	It achieved 84.8% precision and 62.8% success rate on the OTB2013 dataset.
Zhang et al. [29]	The approach employs a hierarchical correlation filter model (HCFM) based on multi-level convolutional features for parallel target tracking.	This model introduces an adaptive update strategy for DAM and HCFM to accommodate target appearance changes.	It relies on color histogram information to generate masks, sensitive to variations in lighting, occlusions, or changes in target appearance.	This approach achieved 93.5% accuracy and 70.8% success rate on the OTB2013 dataset.
Moorthy et al. [30]	The method employs correlation filters based on Gaussian distribution to estimate target position, using kernel ridge regression to mitigate tracking failures.	It employs adaptive scale estimation to detect target size variations and uses dual-feature integration to enhance filter discriminability.	The approach represents targets using feature descriptors, with changes in appearance potentially diminishing tracking performance.	GCF achieved a precision of 86.3% and a success rate of 59.3% on the OTB2013 dataset.
Xia et al. [31]	This approach introduces an occlusion to the KCF, employing an enhanced algorithm based on the Unscented Rauch–Tung–Striebel Smoother when targets are occluded.	The model integrates color histograms with KCF and incorporates sparse representation into training, enhancing algorithmic stability.	It aims to streamline its objective function and identify more efficient model fusion strategies to enhance accuracy.	The method maintains robust performance under various disturbances, averaging 55.2 FPS.
Yan et al. [32]	This method employs a Top-hat filter for target area enhancement, detects small candidate regions via gray-level region growing, and utilizes threshold segmentation for final target identification.	It employs KCF to identify outliers in response maps and uses Kalman filtering at peak anomalies to predict target trajectories, with tracking accuracy notably enhanced.	The Kalman-filter-based KCF algorithm exhibits a slight reduction in tracking speed.	This approach sustains a performance of 24.9 FPS on the dataset, while securing a high accuracy rate of 96.99%.

**Table 3:** Assessment of test outcomes.

Correlation Filter Algorithms	Assessment
BOOSTING Tracker	A stable algorithm with over a decade of application, yet it does not demonstrate significant advantages compared to similar advanced trackers like MIL and KCF.
MIL Tracker	Prone to drifting and incapable of recovering from tracking losses.
KCF Tracker	Exhibits both high accuracy and speed, surpassing MIL in accurately determining tracking failures.
TLD Tracker	Capable of managing occlusions, but its stability is compromised and it is prone to interference from similar-looking targets.
MOSSE Tracker	Characterized by its simplicity and remarkable speed, though its performance is moderate.
CSRT Tracker	Distinguished by its high accuracy, but at the cost of reduced speed.

**Table 4:** Experimental configuration.

Experimental Configuration	
CPU	Intel Intelling m9 processor, Intel Corporation, Santa Clara, USA
MIL-Tracker	16 GB RAM
GPU	NVIDIA GeForce RTX 3060, NVIDIA Corporation, Santa Clara, USA
OP	Windows 11
Python	Python 3.9

**Table 5:** Comparison results of the SiamRPN algorithm before and after improvement.

Network	AOR	Precision	Success	L2 Distance
SiamRPN	0.469	0.559	0.434	45.21
SiamRPN-C	0.495	0.583	0.458	43.36

**Table 6:** Performance comparison of algorithms on the OTB2015 dataset.

Tracker	Success	Precision
DSiam-CnK	0.921	0.909
MIL-Tracker	0.901	0.882
SiamRPN	0.884	0.811
SiamFC	0.821	0.805
CFnet	0.805	0.749
KCF	0.702	0.676
SiamR-CNN [34]	0.701	0.891
SiamTPR [35]	0.702	0.902
SwinTrack [36]	0.691	0.902

**Table 7:** Performance comparison of algorithms on the VOT2018 dataset.

Tracker	A	R	EAO
DSiam-CnK	0.467	1.353	0.264
MIL-Tracker	0.455	1.017	0.231
SiamRPN	0.398	0.831	0.165
SiamFC	0.428	1.110	0.237
CFnet	0.450	1.070	0.188
KCF	0.372	0.978	0.161

**Table 8:** Performance comparison between SiamRPN and DSiam-CnK.

Tracker	SiamRPN	DSiam-CnK	Performance Enhancement
OTB-success	0.901	0.921	2.22%
OTB-precision	0.882	0.909	3.0%
VOT-A	0.455	0.467	2.63%
VOT-R	1.017	1.353	33.04%
VOT-EAO	0.231	0.264	14.29%

**Table 9:** Performance comparison of algorithms on the LaSOT dataset.

Tracker	Precision	Normalized Precision	Success
DSiam-CnK	0.353	0.344	0.390
ECO	0.301	0.338	0.324
DSiam	0.322	0.405	0.333
SiamFC	0.339	0.420	0.336
CFnet	0.259	0.312	0.275
KCF	0.166	0.190	0.178
MDNet	0.373	0.460	0.397

## Data Availability

The original contributions presented in the study are included in the article; further inquiries can be directed to the corresponding authors.
